# Properties of novel PBC/κ-carrageenan hydrogels: probiotic bacterial cellulose synthesized by co-culture of *K. xylinus* with *L. plantarum* or *P. pentosaceus*

**DOI:** 10.3389/fbioe.2026.1914774

**Published:** 2026-09-03

**Authors:** Mainak Chaudhuri, Nabanita Saha, Oyunchimeg Zandraa, Arita Dubnika, Karina Egle, Inga Jurgelane, Petr Saha

**Affiliations:** 1 Centre of Polymer Systems, Tomas Bata University in Zlin, Zlin, Czechia; 2 Institute of Biomaterials and Bioengineering, Faculty of Natural Sciences and Technology, Riga Technical University, Riga, Latvia; 3 Baltic Biomaterials Centre of Excellence, Headquarters at Riga Technical University, Riga, Latvia

**Keywords:** antimicrobial, biocompatible, biomaterial, hydrogel, probiotic bacterial cellulose, wound healing, κ-carrageenan

## Abstract

**Background:**

The development of new hydrogels with antimicrobial, biocompatible, and controlled drug-release properties remains a significant challenge for wound-healing applications. The present study primarily focused on the development of PBC/κ-carrageenan composite hydrogels using two different types of probiotic bacterial cellulose (PBC) named as PBC_LP and PBC_PP, which have not been reported previously.

**Methods:**

In this study, two distinct types of probiotic bacterial cellulose (PBC) were synthesized and designated as (PBC_LP) and (PBC_PP). The synthesis of these two distinct types of PBC was accomplished *via in situ* co-culture of *Komagataeibacter xylinus* with *Lactiplantibacillus plantarum (previously Lactobacillus plantarum)* and *Pediococcus pentosaceus*, separately. “PBC_LP” and “PBC_PP”-based hydrogels were developed by mixing PBCs with κ-Carrageenan using physical and potassium-ion-mediated ionic crosslinking methodologies. The bacterial cellulose (BC)-based hydrogel was selected as a control group for the experimental investigation.

**Results:**

Structural characterization by FTIR and XRD confirmed successful integration of BC as well as PBC with κ-Carrageenan through physical and ionic interactions. SEM analysis revealed highly porous, interconnected three-dimensional architectures, which are favourable for biomedical applications. The hydrogels exhibited high porosity, high equilibrium liquid content (>95%), and rapid swelling within 30 min. Ionic crosslinking has been demonstrated to enhance gel fraction and rheological stability, while reducing pore size, porosity, and swelling behavior. Rheological analysis demonstrated stable viscoelastic gel-like behavior and shear-thinning properties, suitable for injectable or adaptable wound-healing materials. *In vitro* cytocompatibility studies using BALB/3T3 fibroblast cells demonstrated favourable cell viability, particularly for non-crosslinked PBC-based hydrogels, indicating favourable cellular interactions and proliferation. Furthermore, the hydrogels exhibited efficient uptake and a biphasic release profile for Betadine and Gentian Violet, with crosslinked systems showing a more controlled release profile. Importantly, PBC-containing hydrogels exhibited inhibitory activity against *Escherichia coli*, *Staphylococcus aureus*, and *Candida albicans*, which was further enhanced upon loading with antimicrobial agents.

**Conclusion:**

The PBC/κ-Carrageenan hydrogels developed in this study exhibit a combination of advantageous structural, biological, rheological, antimicrobial, and controlled-release properties. This underscores their potential as multifunctional wound-healing and antimicrobial-delivery systems, making the hydrogel a potential candidate for tissue-engineering and wound-healing applications after *in vivo* and clinical studies in future work.

## 1 Introduction

Nowadays, infected wounds are a major clinical challenge worldwide, causing delayed healing, chronic inflammation, microbial colonization and increasing antimicrobial resistance. Effective wound management requires biomaterials that can maintain a moist environment (Stoica et al., 2020), promote cell division (Kibungu et al., 2021; Sawadkar et al., 2025), prevent infection (Vivcharenko and Przekora, 2021; Ghosh et al., 2024) and support the controlled delivery of therapeutics (Ghosh et al., 2024). Hydrogel-based wound healing materials have attracted significant attention due to their high water content, extracellular matrix (ECM)-mimicking structure, oxygen and nutrient permeability, and ability to deliver bioactive components (Das et al., 2021; Zhang et al., 2024). Naturally derived polysaccharide-based biomaterials are particularly attractive among the various hydrogel systems due to their good biocompatibility, biodegradability, low toxicity, and structural similarity to natural tissues (Sharma et al., 2024).

Bacterial cellulose (BC), a polysaccharide that is naturally synthesized by bacteria such as *Komagataeibacter xylinus* (*Komagataeibacter xylinus*), has emerged as a promising biomaterial for use in wound healing and tissue engineering. BC has a unique three-dimensional nanofibrous structure, is highly crystalline, has excellent mechanical strength and improved water retention capacity, and is remarkably biocompatible (Ma et al., 2020; Wu et al., 2023). These properties make it highly suitable for various biomedical applications, including wound dressings, drug delivery systems, scaffolds and artificial tissues (Carvalho et al., 2019; Liu et al., 2020; De Amorim et al., 2022). However, pure BC has some limitations despite its advantageous properties, such as a lack of antimicrobial activity, low intrinsic bioactivity, and insufficient flexibility for certain biomedical applications. Consequently, numerous studies have focused on modifying or combining BC with complementary polymers to enhance its biological and physicochemical performance (Eslahi et al., 2020; Saha et al., 2025).

Probiotic-derived bacterial cellulose (PBC) has recently attracted significant research interest as a next-generation biomaterial with enhanced functionality (Sabio et al., 2021; Chaudhuri et al., 2025). Cultivating cellulose-producing bacteria alongside probiotic microorganisms in an *in situ* co-culture system can confer additional bioactive properties to the cellulose matrix, including antimicrobial activity and enhanced cellular interactions (Sabio et al., 2021; Chaudhuri et al., 2026). Probiotic strains such as *Lactiplantibacillus plantarum (Lactiplantibacillus plantarum)* and *P. pentosaceus (Pediococcus pentosaceus)* produce antimicrobial metabolites, organic acids and bacteriocins, which are commonly capable of inhibiting the pathogens involved in wound infections (Kahraman et al., 2026). Therefore, incorporating these probiotic-derived components into cellulose-based biomaterials could be an innovative way to enhance both antimicrobial efficacy and cytocompatibility, reducing reliance on synthetic antimicrobial agents.

In addition to BC, the sulfated polysaccharide κ-Carrageenan (κ-C), which is derived from red seaweed, has been the subject of extensive research in relation to its use in the preparation of hydrogels. This is because of its hydrophilicity, biocompatibility, gelling efficiency, and ionic crosslinking behaviour (Kumar et al., 2025). The sulfate groups present in κ-Carrageenan facilitate the formation of physically crosslinked hydrogels in the presence of monovalent cations, such as potassium ions (K^+^), which enable the formation of mechanically stable and highly hydrated polymeric networks (Kim and Lee, 2016). In addition, κ-Carrageenan-based hydrogels exhibit favourable swelling behaviour, controllable porosity, and the ability to bind and release therapeutic compounds in a controlled manner, rendering them suitable candidates for wound healing and drug delivery (Qamar et al., 2024). However, κ-Carrageenan hydrogels alone often exhibit limited mechanical strength and structural stability, requiring their incorporation into reinforcing biopolymers.

The combination of BC with κ-Carrageenan has been identified as a promising approach to fabricate multifunctional hydrogels with synergistically enhanced properties (Ngwabebhoh et al., 2021; Botleng et al., 2024). The nanofibrous cellulose network has been demonstrated to enhance the hydrogel matrix, thereby improving structural integrity (Wang et al., 2021). In addition, κ-Carrageenan has been shown to contribute to flexibility, hydrophilicity, and ionic reactivity (Jabeen et al., 2025; Kumar et al., 2025). Moreover, the incorporation of probiotic-derived cellulose has been shown to confer intrinsic antimicrobial activity and enhance cellular compatibility (Chaudhuri et al., 2026). Such multifunctional hydrogel systems are of particular interest in wound healing, where an ideal dressing would simultaneously support tissue regeneration, absorb wound exudate, maintain moisture balance, prevent microbial contamination, and ensure a sustained supply of antimicrobial agents.

Even though several BC/κ-Carrageenan composite hydrogels have been documented in the literature, research on the incorporation of PBC into κ-Carrageenan matrices remains limited. Our previous study discussed the preparation and physicochemical properties of the PBC_LpPp/κ-Carrageenan hydrogel and its beneficial effects for wound-healing applications (Chaudhuri et al., 2026). The PBC_LpPp was synthesized by co-culture of *K. xylinus* with *L. plantarum* and *P. pentosaceus* together. However, the effects of hydrogels prepared from κ-Carrageenan and probiotic-derived cellulose obtained by *in situ* co-culture *of K. xylinus* with *L. plantarum* and *P. pentosaceus*, individually (PBC_LP/κ-Carrageenan and PBC_LP/κ-Carrageenan), on structural, physicochemical, rheological, cell-compatibility, and antimicrobial performance have not yet been extensively investigated. Furthermore, the potential of these hydrogels as antimicrobial solution delivery systems, as well as their ability to control the release of agents such as betadine and gentian violet (GV), has not yet been extensively explored. The novelty of this work lies in the preparation of different types of PBC/κ-Carrageenan hydrogels (using PBC_LP and PBC_PP) and in the comparative study of their structural and functional properties, suitable for wound-healing applications.

**SCHEME 1:**
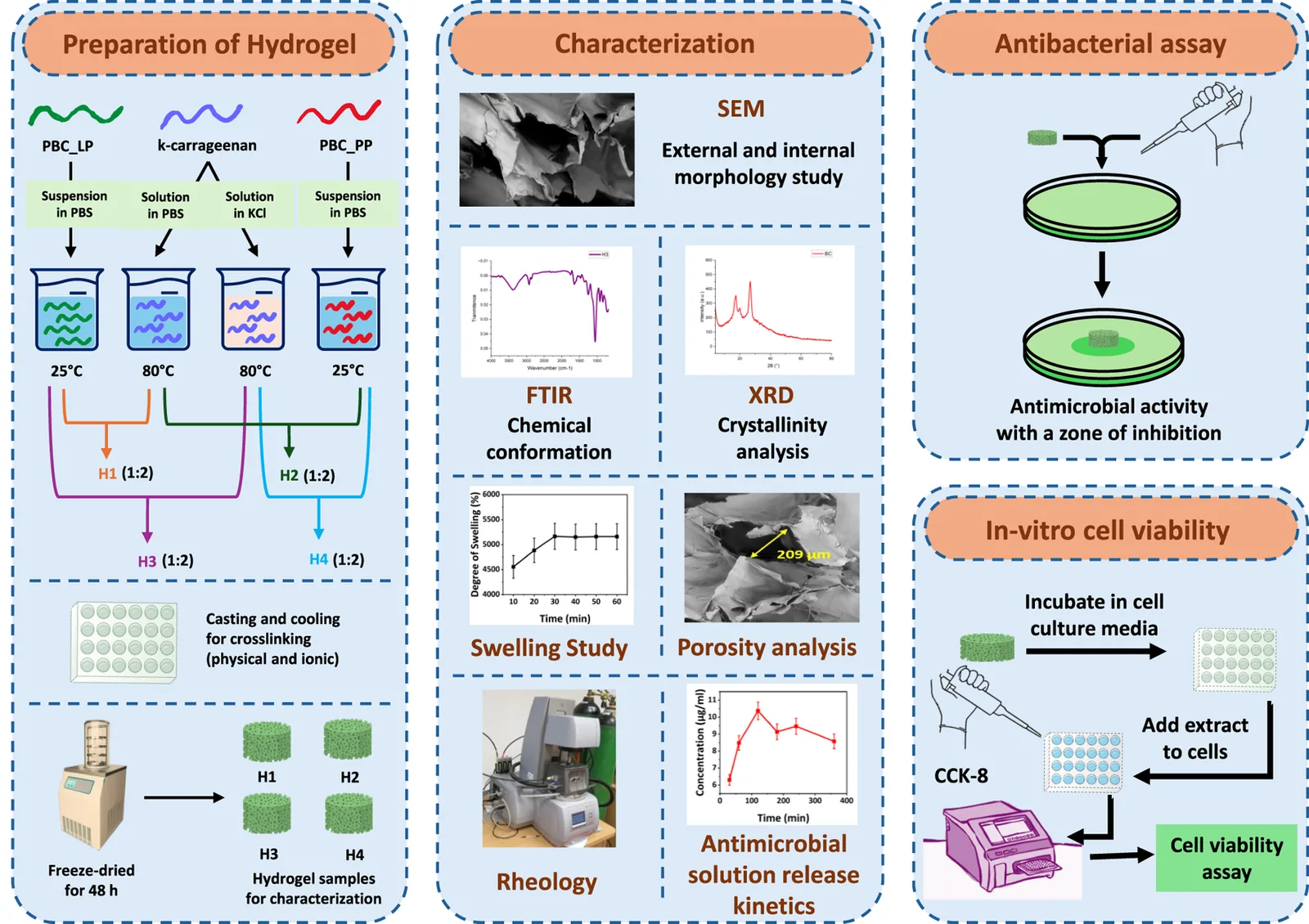
Schematic Diagram of preparation, characterization, antimicrobial and cell-viability assay of PBC/κ-Carrageenan hydrogel.

In the present study, multifunctional PBC/κ-Carrageenan composite hydrogels were prepared using two different PBCs (synthesized by co-culturing *K. xylinus* and *L. plantarum*: PBC_LP, and *K. xylinus* and *P. pentosaceus*: PBC_PP) separately with κ-Carrageenan. The investigation involved the preparation and comparative analysis of hydrogels, with and without potassium ion-mediated ionic crosslinking, with the objective of evaluating the impact of their physicochemical and biological performance. The developed hydrogels were systematically characterized in terms of structural composition, crystallinity, shape, porosity, swelling behaviour, gel ratio, flowability, cell-compatibility, antimicrobial solute absorption and time-dependent release profile. Moreover, the antimicrobial efficacy against *E. coli (Escherichia coli), S. aureus (Staphylococcus aureus),* and *C. albicans (Candida albicans)* were also evaluated. The results of this work reveal the potential of PBC/κ-Carrageenan hydrogels as versatile biomaterials for wound healing and antimicrobial agent delivery applications. Scheme 1 represents an overview of the whole study.

## 2 Materials and methods

### 2.1 Materials

PBC_LP and PBC_PP were synthesised in the laboratory of the Centre of Polymer Systems (CPS), Tomas Bata University in Zlin, Czech Republic. The PBC_LP (8.01 ± 0.57 g/L) was synthesized by co-culture of *K. xylinus* and *L. Plantarum* in AJMRS medium (apple juice: MRS medium = 1:1) by incubating for 14 days at 30 °C. Similarly, the PBC_PP (7.48 ± 1.51 g/L) was synthesized using the same culture condition, but *P*. *pentosaceus* was used instead of *L. plantarum*. Absolute ethanol (EtOH), κ-Carrageenan and Potassium Chloride (KCl) were supplied by Sigma-Aldrich, Czech Republic. Gentian Violet (GV) and Betadine were purchased from a local pharmacy. Distilled water was used for the preparation of all the solutions. Cytotoxicity test was conducted by using CCK-8 kit (Sigma Aldrich, St. Louis, USA) along with a complete cell medium, which consist of 10% calf serum (Sigma Aldrich, St. Louis, USA), 1% penicillin-streptomycin (Sigma Aldrich, St. Louis, USA), and 89% of Dulbecco’s modified Eagle’s medium (DMEM) (Sigma Aldrich, St. Louis, USA).

### 2.2 Preparation of PBC/κ-carrageenan-based hydrogels

PBC/κ-Carrageenan-based hydrogels were developed using two different types of PBC suspension (1%W/V) (PBC_LP and PBC_PP) and κ-Carrageenan solution (2% W/V), both without using any crosslinker and with KCl as ionic crosslinker.

PBC_LP and PBC_PP were synthesized in the lab of the Centre of Polymer Systems, Tomas Bata University in Zlin, Czech Republic, by co-culturing *K. xylinus* individually *with L. plantarum* and *P. pentosaceus*, respectively. Thereafter, a homogeneous suspension (in PBS) of PBC_LP and PBC_PP (1% W/V) was made at room temperature (25 °C) by blending both types of PBC mat using the Kilig Vortex Blender (Kilig, Bengaluru, India) for 2 min at 22,000 rpm. The blending process was performed only for 2 min to obtain a homogeneous cellulose suspension suitable for hydrogel preparation while minimizing mechanical stress to the embedded probiotic cells. Although high-shear homogenization may reduce bacterial viability, the viability of probiotic cells after blending was not quantified in the present study, as the goal was to introduce inhibitory activity (derived from probiotic bacteria or their derivatives) in the hydrogel against *E. coli and S. aureus*.

κ-Carrageenan solution (2% W/V) of two different types was prepared for hydrogel preparation. The first solution was prepared by dissolving κ-Carrageenan into DI, water (80 °C). Whereas, in order to prepare the second κ-Carrageenan solution, a 20 mM KCl, solution was used instead of DI, water.

Four different types of hydrogels were prepared by mixing (at room temperature for 2 min) two PBC suspensions with κ-Carrageenan solutions to form homogeneous mixtures, which were then allowed to crosslink at room temperature. The prepared hydrogels were; PBC_LP: κ-Carrageenan (without KCl) = 1:2 (H1), PBC_PP: κ-Carrageenan (without KCl) = 1:2 (H2), PBC_LP: κ-Carrageenan (with KCl) = 1:2 (H3) and PBC_PP: κ-Carrageenan (with KCl) = 1:2 (H4). Two control hydrogel samples were prepared: BC: κ-Carrageenan (without KCl) = 1:2 (X1), and BC: κ-Carrageenan (with KCl) = 1:2 (X2). The viability of probiotic cells was not assessed after the hydrogel preparation. Table 1 presents the formulations and mixing ratios of all the hydrogel samples. All the hydrogels were freeze-dried at −110 °C for 24 h using a freeze-dryer (Labogene ScanVac CoolSafe Trigon plus) and stored in a desiccator for further characterizations (Nguyen et al., 2021; Chaudhuri et al., 2026).

**TABLE 1:** Composition of BC/κ-Carrageenan, PBC_LP/κ-Carrageenan and PBC_PP/κ-Carrageenan hydrogel with mixing ratio of polymers.

​	Sample index	Biopolymers			
		*BC*	*PBC_LP*	*PBC_PP*	*κ-Carrageenan*
Without KCl	X1	1	0	0	2
	H1	0	1	0	2
	H2	0	0	1	2
With KCl	X2	1	0	0	2
	H3	0	1	0	2
	H4	0	0	1	2

### 2.3 Characterization

#### 2.3.1 Structural analysis

The functional groups in all the hydrogel samples were identified by analyzing the Fourier transform infrared (FTIR) spectra recorded between the scanning range of 4,000 and 400 cm^-1^ at a resolution of 4.0 cm^-1^ using a Nicolet iS5 (Thermo Scientific, USA) (Saha et al., 2025; Chaudhuri et al., 2026).

The surface and internal morphology and microstructure of PBC_LP/κ-Carrageenan and PBC_PP/κ-Carrageenan hydrogels were analysed using a Scanning Electron Microscope (FEI, Brno, Czech Republic) with an accelerating voltage of 5 kV. The freeze-dried hydrogel samples were coated with Au/Pd using a fine coater (JEOL JFC 1300, Tokyo, Japan) for the improvement of surface conductivity (Nguyen et al., 2021; Chaudhuri et al., 2026).

The crystalline structure of the PBC_LP/κ-Carrageenan and PBC_PP/κ-Carrageenan hydrogels was measured by using an X-ray diffractometer (MiniFlex 600, Rigaku, USA). The measurements were made using the CoKα radiation (λ = 1.79 Å) at 15 mA and 40 kV. The 2θ values were measured between 4° and 80°. Thereafter, the data were transformed to CuKα (λ = 0.1542 nm) using a PowDLL converter (version 2.911.0.0) (Challa et al., 2025). The crystalline index (%) of analysed samples was calculated by dividing the diffractogram area due to the crystalline region by the total area of the original diffractogram using equation (Nguyen et al., 2021; Salem et al., 2023) (Eqaution 1):

$$\text{Crystallinity index }\left(\text{CI}\right)\;\%=\frac{\text{Area}\;\text{of}\;\text{all}\;\text{the}\;\text{crystalline}\;\text{peaks}}{\text{Area}\;\text{of}\;\text{all}\;\text{the}\;\text{crystalline}\;\text{and}\;\text{amorphous}\;\text{peaks}}\times 100$$ (1)

#### 2.3.2 Porosity

The percentage of porosity of the PBC_LP/κ-Carrageenan and PBC_PP/κ-Carrageenan hydrogels were measured to evaluate the total amount of pores in the hydrogel internal structure by the liquid-displacement method (Basu et al., 2019). All hydrogels [thickness = 3–5mm, diameter = 14 mm] were allowed to soak in absolute ethanol at 37 °C for 2 h, until the saturation point. The porosity (%) of the hydrogels was measured by Equation 2:

$$\text{Porosity }\left(\%\right)=\left(W_{2}\;‐W_{1}\right)/\rho V_{1}$$ (2)

Where W_1_ and W_2_ are denoted as the weights of the hydrogels measured before and after immersion in absolute ethanol, respectively. V_1_ is the volume (V_1_ = πr^2^h, where r is the radius and h is the thickness of the sample, as all samples are cylindrical in shape) of the hydrogels before their immersion in ethanol, and ρ (0.00078691 g/mm^3^ at 37 °C) is the density constant of ethanol (Chaudhuri et al., 2026). The results were measured in triplicate, and the average value is reported.

The internal pore size of the hydrogels was measured using cross-section SEM images. For each hydrogel sample, measurements were performed 5 times, and the average value is demonstrated as the result.

#### 2.3.3 Swelling study

The swelling analysis was conducted at 37 °C to assess the swelling behaviour of the hydrogel samples in phosphate buffer solution (PBS) (pH 7.4). The freeze-dried hydrogels were allowed to soak in PBS solution, and the swell weight was recorded at specific time intermissions (10, 20, 30, 40, 50, and 60 min) until their optimum swelling. The degree of swelling was calculated following Equation 3:

$$\text{Degree of swelling }\left(\%\right)=\left[\left(W_{s}\;–\;W_{d}\right)/\;W_{d}\right]\times 100$$ (3)

where W_s_ and W_d_ are the swollen and the dry weights of the hydrogels, respectively (Saha et al., 2025; Chaudhuri et al., 2026).

The equilibrium liquid content (ELC) of the hydrogels was measured when the swelling reached equilibrium and calculated by Equation 4:

$$\text{ELC }\left(\%\right)=\left[\left(W_{s}\;‐\;W_{0}\right)\;/\;W_{s}\right]\times 100$$ (4)

where W_0_ and W_s_ are the initial dry weight and the swollen weight of the hydrogel samples at equilibrium, respectively (Saha et al., 2025; Chaudhuri et al., 2026).

The fully swollen hydrogels were dried in a vacuum oven until a constant weight was reached. Thereafter, the dried hydrogel samples were reweighted, and the gel fraction was calculated using Equation 5:

$$\text{Gel fraction }\left(\%\right)=\left(M_{d}/M_{0}\right)\times 100$$ (5)

Where M_0_ and M_d_ are the initial dry weight and second dry weight (the dry weight of hydrogels after swelling) of the hydrogels, respectively (Wong et al., 2015; Chaudhuri et al., 2026).

All results were measured in triplicate, and the average value is reported.

#### 2.3.4 *In vitro* cell viability

The cell viability of the PBC_LP/κ-Carrageenan and PBC_PP/κ-Carrageenan hydrogels was evaluated by using murine fibroblast cells (BALB/3T3) *via* an indirect extraction method. This experiment was conducted at the Baltic Biomaterials Centre of Excellence (BBCE, Riga, Latvia). First, the hydrogels were sterilized (using UV light) for a duration of 30 min. After the sterilised hydrogels (50 mg/mL) were subjected to an incubation process in Dulbecco’s Modified Eagle Medium (DMEM), a solution that was enriched with 10% calf serum and 1% penicillin/streptomycin. This procedure was undertaken to yield hydrogel extracts. Following 24 and 48 h of culture in the medium at 37 °C, the hydrogel extracts were collected and kept at −20 °C until further analysis.

Standard conditions (a humidified atmosphere, 37 °C, 5% CO_2_) were utilized to cultivate BALB/3T3 cells. Once the cells reached approximately 80% confluence, trypsin was employed to disaggregate them, and subsequently, they were washed with Phosphate Buffered Saline (PBS). Following a thorough enumeration process, the cells were subsequently cultivated at a density of 5 × 10^3^ cells per well within 96-well plates. These plates were then placed in a humidified incubator, where the cells were permitted to adhere and proliferate overnight. After cell attachment, the hydrogel extracts were used to replace the culture medium. Subsequently, the plates were incubated for 24 h at 37 °C. Thereafter, 10 μL of CCK-8 was added to each well, comprising 100 μL of culture media, and the plate was incubated for 2 h. The absorbance was measured at 450 nm wavelength using a microplate reader. Cell viability was expressed as a percentage relative to the control (cells grown in growth medium instead of hydrogel extract) (Chaudhuri et al., 2026). All results are measured in triplicate and reported as average values.

#### 2.3.5 Rheological assay

A rotational rheometer (Anton Paar MCR 502) with a 25 mm parallel plate geometry and a constant temperature of 37 °C was utilized to perform rheological experiments. A strain sweep test was initially conducted over a strain range of 0.1%–100% at a fixed frequency of 1 Hz to ascertain the linear viscoelastic (LVE) area. Subsequently, a frequency-sweep experiment was performed, varying the angular frequency from 0.1 to 100 rad/s while maintaining a constant strain of 1%. A comprehensive evaluation was undertaken, encompassing the measurement of storage modulus (G′), loss modulus (G″), and complex viscosity (η*), for all the hydrogel samples (X1 -H4) (Saha et al., 2025).

#### 2.3.6 Antimicrobial solution uptake and *in vitro* release profile

The antimicrobial solution uptake efficiency of the freeze-dried hydrogels (X1-H4) was evaluated by measuring the absorption of antimicrobial solutions by the hydrogel samples. Two different antimicrobial solutions were prepared by diluting Betadine and Gentian Violet (GV) separately in a 1:10 ratio with DI water.

All the freeze-dried hydrogel samples (X1-H4) were allowed to soak in Betadine and GV solutions for 10 min. The betadine-soaked samples were named as X1_B_, X2_B_, H1_B_, H2_B_, H3_B_, H4_B_; and the GV-soaked samples were named as X1_GV_, X2_GV_, H1_GV_, H2_GV_, H3_GV_, H4_GV_.

The antimicrobial solution loading capacity of the freeze-dried hydrogels was then calculated by using the following Equation 6.

$$\text{Loading capacity }\left(g/g\right)=\left(W_{s}-W_{d}\right)\;/\;W_{d}$$ (6)

where W_d_ and W_s_ are the weights of the dried and swollen hydrogels after soaking the betadine and GV solutions, respectively (Chaudhuri et al., 2026).

Antimicrobial liquid (Betadine and GV) release profile was assayed using the betadine-soaked (X1_B_- H4_B_) and GV-soaked (X1_GV_- H4_GV_) samples. All the antimicrobial solution-soaked hydrogel samples were placed in 20 mL of PBS, and the betadine and GV were allowed to be released from the hydrogel network into the PBS solution separately. 2 mL of PBS was collected at specific time intervals (30, 60, 120, 180, 240 and 360 min) and kept for UV-vis spectrophotometric analysis. To maintain the sink condition, 2 mL of PBS was added to each beaker immediately after collecting the samples. All the samples were measured at 288 nm (Garg et al., 2007; Kida et al., 2020) nm for betadine and 591 nm (Abbas et al., 2019) for GV using a UV-Vis spectrophotometer (SPECORD 250 Plus, analytic Jena AG, Germany).

The concentrations of betadine and GV in the PBS solution at each time point were measured using a standard curve generated by linear regression analysis. To prepare the standard curves for betadine and GV, working stock solutions were prepared for both. The working stock solution of Betadine was prepared by diluting it 1:100 with PBS. Serial dilutions of Betadine working stock solution were prepared using 0.5, 1.0, 1.5, 2.0, and 2.5 mL, then the final volumes were adjusted to 10 mL by adding PBS. The absorbance of each betadine solution was measured at 288 nm (Garg et al., 2007), using PBS as a blank. The working stock solution of GV was prepared by adding 100 μL of GV to 100 mL of PBS. Then serial dilutions of the GV working stock solution were prepared using 0.5, 1.0, 1.5, 2.0, and 2.5 mL of GV, and similarly, the final volume was adjusted to 10 mL by adding PBS. The absorbance of each GV solution was measured at 591 nm (Abbas et al., 2019), using PBS as a blank. Thereafter, standard curves for both betadine and GV were prepared by plotting absorbance vs. concentration.

#### 2.3.7 Antimicrobial assay

The antibacterial activity of PBC_LP/κ-Carrageenan and PBC_PP/κ-Carrageenan hydrogels (freeze-dried, betadine-soaked, and gentian violet-soaked) was investigated against pathogenic bacteria *E. coli* (Gram-negative), *S. aureus* (Gram-positive), and fungi *C. albicans* using the agar disc diffusion method. An aliquot of each bacterial stock (amount: 0.1 mL and concentration 14 × 10^8^ CFU/mL) was spread uniformly on TSA plates for bacteria and SDMA plates for fungi. The freeze-dried, betadine-soaked, and GV-soaked hydrogel samples were cut into disc-shaped (14 mm diameter) samples. Thereafter, the hydrogel samples were placed on agar plates with the bacterial suspension. The antimicrobial activity was evaluated after incubation for 24 h at 37 °C, by measuring the zone of inhibition formed around the hydrogel samples.

## 3 Results and discussion

### 3.1 Hydrogel formation and physical appearance

**FIGURE 1:**
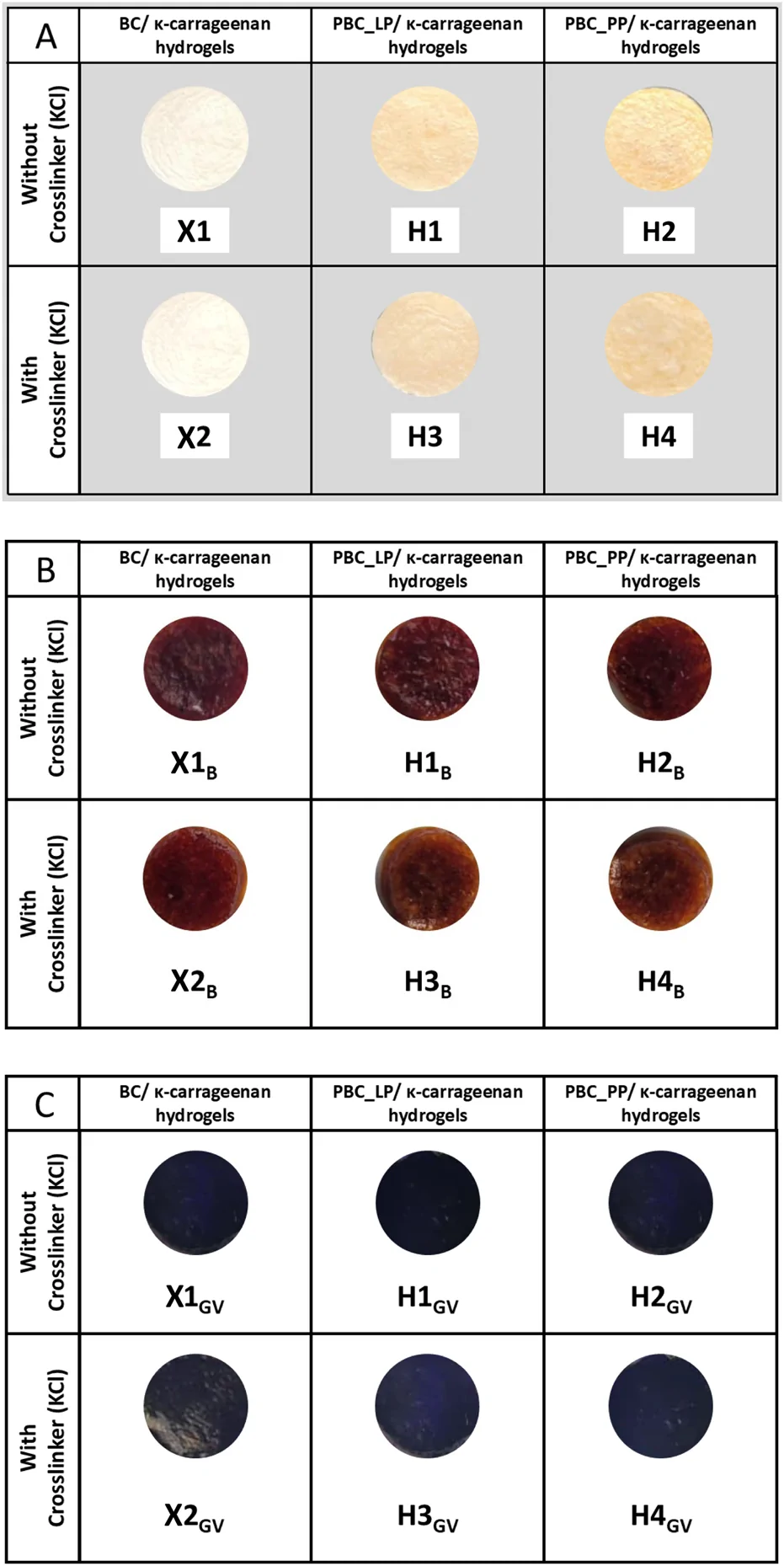
Physical appearance of freeze-dried **(A)**, betadine-soaked **(B)**, and gentian violet-soaked **(C)** BC/κ-carrageenan, PBC_LP/κ-Carrageenan and PBC_PP/κ-Carrageenan hydrogels.

The physical appearance of the freeze-dried PBC_LP/κ-Carrageenan and PBC_PP/κ-Carrageenan hydrogels is demonstrated in Figure 1. Figure 1A demonstrates the images of freeze-dried hydrogels (without Betadine and GV soaked) (X1-H4). The images of Betadine-soaked (X1_B_-H4_B_) and GV-soaked (X1_GV_-H4_GV_) hydrogels are shown in Figures 1B,C, respectively. As can be seen in Figure 1A, the PBC_LP/κ-Carrageenan and PBC_PP/κ-Carrageenan hydrogels (H1- H4) appeared light brown in colour because of the presence of PBC_LP and PBC_PP, and the BC/κ-Carrageenan hydrogels (X1, X2) appeared white due to the presence of normal BC. Figure 1B represents the Betadine-soaked hydrogels, where all the hydrogels turned reddish brown, and the GV-soaked hydrogels dark blue due to the presence of the antimicrobial solutions.

### 3.2 Structural and morphological analysis

**FIGURE 2:**
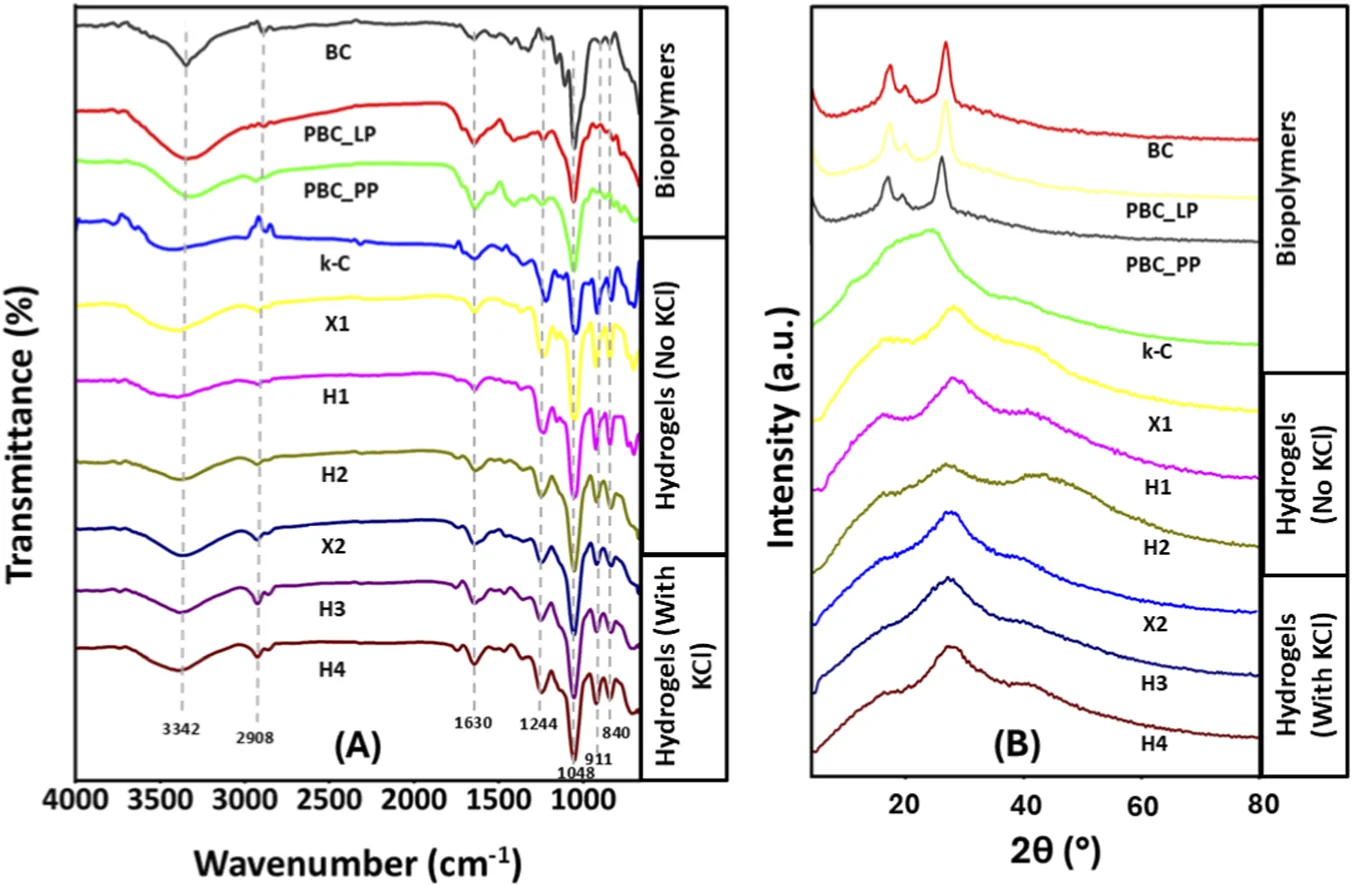
FTIR **(A)** and XRD **(B)** of BC/κ-Carrageenan, PBC_LP/κ-Carrageenan and PBC_PP/κ-Carrageenan hydrogels, where X1: BC/κ-Carrageenan hydrogels (without KCl), H1: PBC_LP/κ-Carrageenan hydrogels (without KCl), H2: PBC_PP/κ-Carrageenan hydrogels (without KCl) and X2: BC/κ-Carrageenan hydrogels (with KCl), H3: PBC_LP/κ-Carrageenan hydrogels (with KCl), H4: PBC_PP/κ-Carrageenan hydrogels (with KCl).

Fourier transform infrared spectroscopy (FTIR) was utilized to investigate the chemical composition and intermolecular interactions of the BC or PBC_LP or PBC_PP with κ-Carrageenan, and the resultant hydrogel composites (X1- H4) (Figure 2A).

The analysis of the samples revealed characteristic bands indicative of the polysaccharide backbones of bacterial cellulose and κ-Carrageenan. The broad absorption band observed at approximately 3,342 cm^-1^ is attributed to O-H stretching vibrations, thereby confirming the presence of extensive intermolecular and molecular hydrogen bonding within the hydrogel matrix (Popescu et al., 2019). A band around 1630 cm^-1^ represents the bending vibration of the absorbed water molecule in the hydrophilic matrix (Shen and Wu, 2003). The peaks located at 2,908 and 1048 cm^-1^ indicate the C-H (Taniguchi and Monde, 2007) and C-O/C-C stretching vibrations of glycosidic bonds (Sekkal et al., 1995), respectively. This phenomenon aligns with the established sugar structure of cellulose and κ-Carrageenan.

Specifically, the incorporation of κ-Carrageenan into the hydrogel network is confirmed by the presence of the critical sulfate ester band. The absorption band at 1244 cm^-1^ is indicative of the S=O stretching vibration of the sulfate group (-OSO_3_^−^) (Pereira et al., 2003; Wang et al., 2025), while the bands at 911 cm^-1^ and 840 cm^-1^ are characteristic of 3,6-anhydro-D-galactose and galactose-4-sulfate (Mendes et al., 2024), respectively. The presence of these peaks in the composite spectra (X1-H4) indicates the successful incorporation of the κ-Carrageenan component into the composite structure.

A comparative analysis of non-crosslinked (X1, H1, H2) and ionically crosslinked (X2, H3, H4) hydrogels reveals that their spectral profiles are largely consistent. The absence of any significant new peaks or major shifts in existing peaks indicates that the hydrogel formation is mainly driven by physical interactions, i.e., hydrogen bonding between the hydroxyl groups of BC/PBC and κ-Carrageenan and electrostatic interactions involving the sulfate groups of κ-Carrageenan. These results substantiate the efficacious fabrication of composite hydrogels through the concerted physical affinity of the components.

The crystalline nature of BC, k-C, and the synthesized hydrogel (X1-H4) was analysed by X-ray diffraction (XRD), as shown in Figure 2B. The XRD pattern of pure BC (red) exhibits intense and well-defined diffraction peaks at approximately 2θ = 14.5°, 16.8°, and 22.7°. These peaks correspond to the $\left(1\bar{1}0\right)$ (110) and (200) crystallographic planes of cellulose *I*, respectively (Wang et al., 2017; Challa et al., 2025), confirming the high degree of crystallinity and highly ordered fibrous structure of bacterial cellulose. In contrast, the κ-Carrageenan spectrum (green) exhibits a broad, diffuse “amorphous halo” centred at 2θ = 20°–25°, which is typical for the partially-crystalline but predominantly disordered arrangement of the carrageenan polymer chains (Jumaidin et al., 2017). During the formation of the composite hydrogels (X1-H4), the characteristic diffraction peaks of bacterial cellulose became broader and less intense than those of normal BC or PBC. This observation indicates a reduction in the apparent crystallinity of the composite hydrogel, which might be attributed to the incorporation of the predominantly amorphous κ-Carrageenan phase and changes in the overall crystalline-amorphous balance of the hydrogel samples. In the non-crosslinked composites (X1, H1, H2), the characteristic BC peaks are still visible but are greatly broadened and weakened. This indicates that the addition of κ-Carrageenan to the bacterial cellulose matrix disrupts the close packing of cellulose microfibrils, thereby reducing the composite system’s overall crystallinity. Interestingly, the introduction of K^+^ ions as crosslinkers in the X2, H3, and H4 samples produces a more amorphous profile. The suppression of the sharp BC diffraction peaks in these samples indicates a more even distribution of cellulose fibres within the κ-Carrageenan matrix, which is likely facilitated by the formation of a dense, ionic crosslinked network, which limits the recrystallisation or self-assembly of cellulose chains. The broad peak observed near 2θ = 28°–30° in the hydrogel samples may be due to structural interference arising from the closely packed, crosslinked polymer network. These results support the FTIR observations, indicating that while the chemical identity of the components remains intact, their physical arrangement has a more disordered, homogeneous phase is often associated with improved water retention and flexibility in biomaterial applications. The crystallinity index of BC, PBC_LP, PBC_PP, κ-Carrageenan and the hydrogel samples (X1-H4) are shown in Table 2.

**TABLE 2:** Crystallinity index of biopolymers and hydrogel samples.

Sample index		Crystallinity index (CI) %
Biopolymers	BC	66.92
	PBC_LP	59.68
	PBC_PP	62.15
	κ-Carrageenan	24.47
Hydrogel samples	X1	28.77
	H1	26.14
	H2	27.63
	X2	26.49
	H3	24.37
	H4	25.81

**FIGURE 3:**
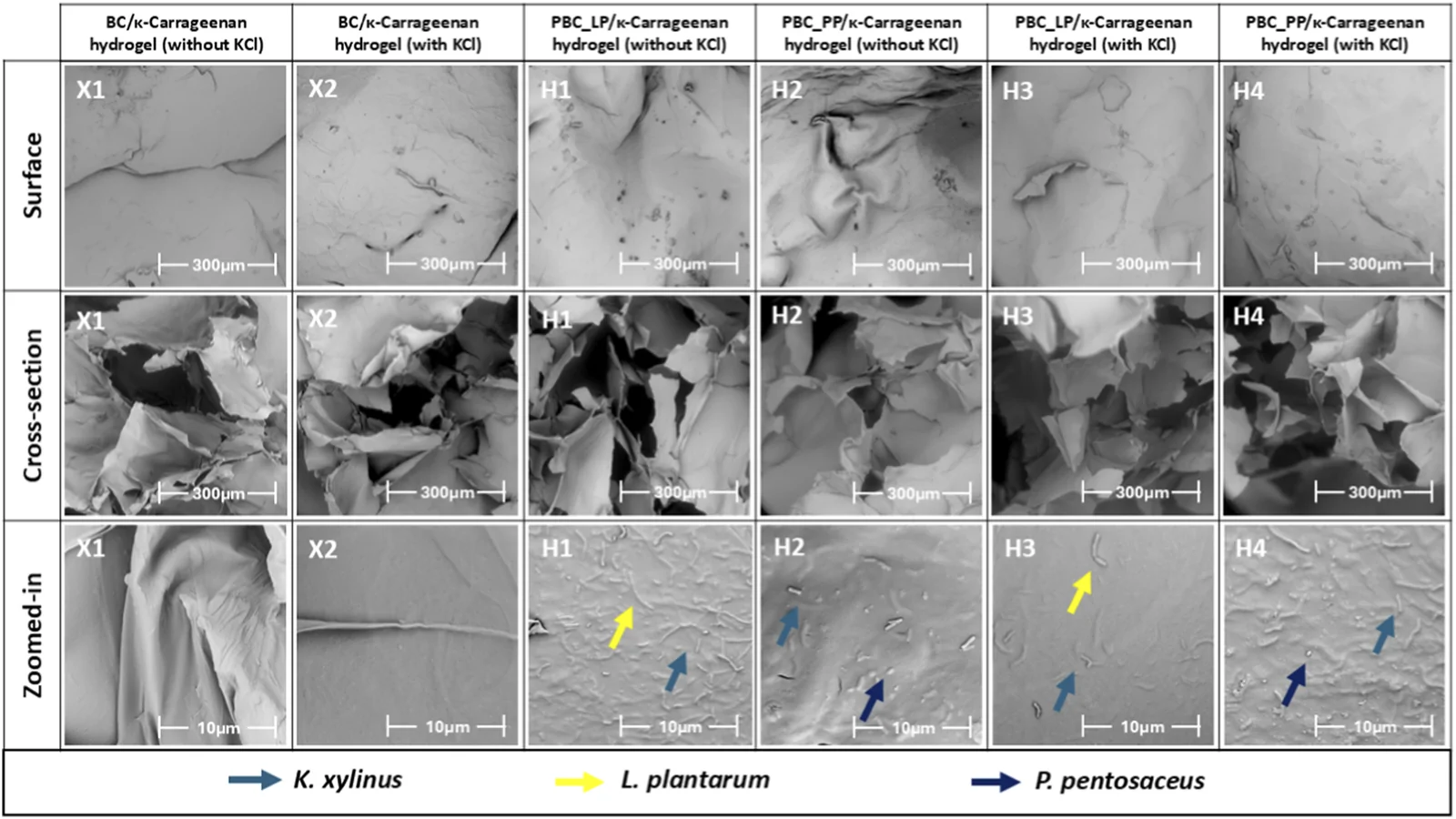
SEM micrographs (surface view, cross-section view and zoomed view) of BC/κ-Carrageenan, PBC_LP/κ-Carrageenan and PBC_PP/κ-Carrageenan based hydrogels.

SEM is used to analyse the surface morphology and microstructure of the freeze-dried hydrogels (X1-H4) by observing the surface and cross-section images, which can be seen in Figure 3. No pores are visible in the surface view of any of the hydrogels. However, the 3D porous network structure of the hydrogel is visible in the cross-sectional view. In the zoomed-in view of the H1 and H3 samples, both the *K. xylinus* and *Lactiplantibacillus palntarum* can be seen, which are derived from the PBC_LP. Moreover, *K. xylinus* and *P. pentosaceus*, derived from the PBC_PP, are visible in the zoomed-in view of the H2 and H4 hydrogel samples. However, no bacterial presence is observed in the X1 and X2 hydrogel samples, as they are prepared with BC and κ-Carrageenan. This porous structure of the hydrogels provides a favourable environment for cell migration and proliferation, making the hydrogels a potential biomaterial for wound healing.

### 3.3 Porosity analysis

**FIGURE 4:**
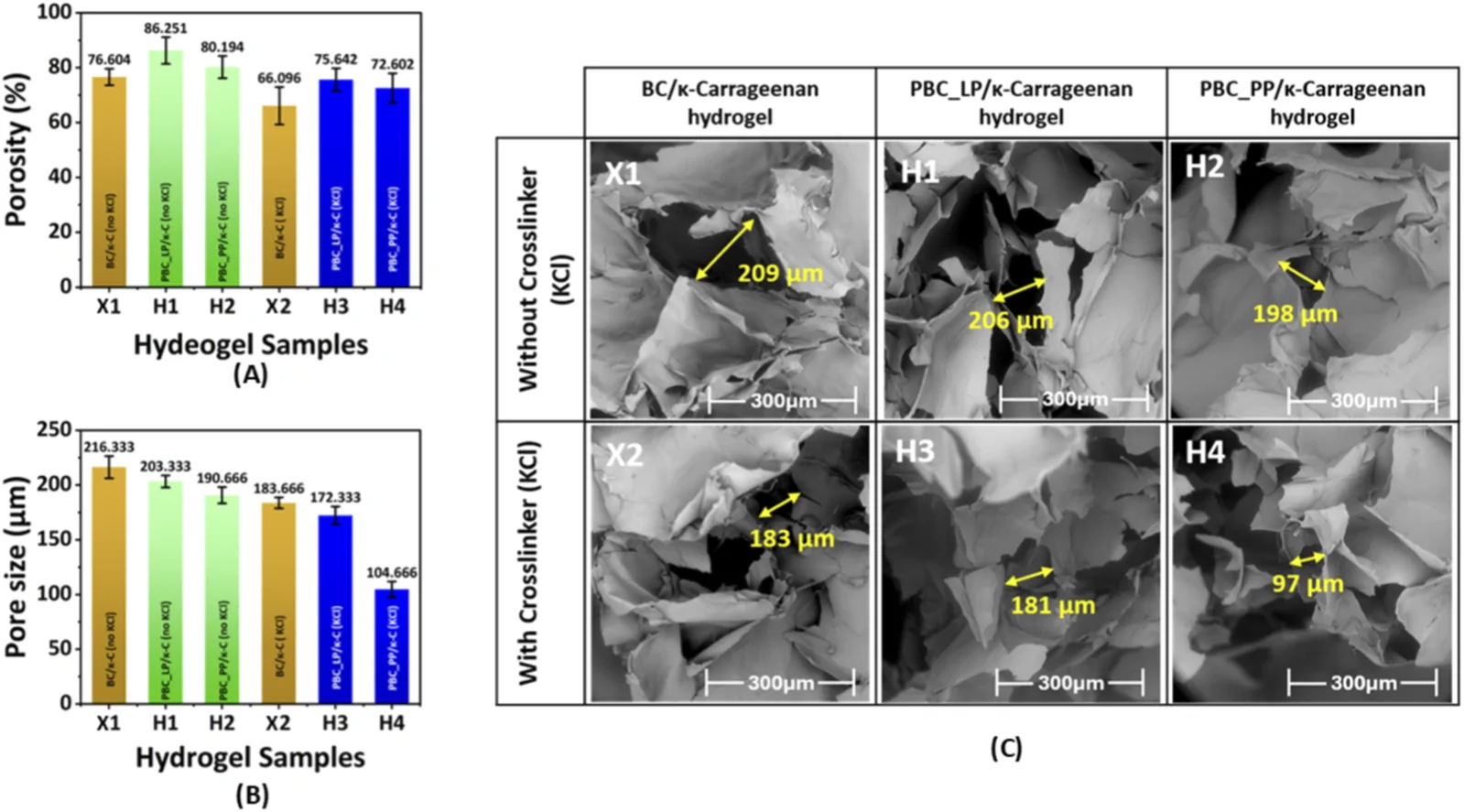
Porosity of BC/κ-Carrageenan, PBC_LP/κ-Carrageenan and PBC_PP/κ-Carrageenan hydrogels, where X1: BC/κ-Carrageenan hydrogels (without KCl), H1: PBC_LP/κ-Carrageenan hydrogels (without KCl), H2: PBC_PP/κ-Carrageenan hydrogels (without KCl) and X2: BC/κ-Carrageenan hydrogels (with KCl), H3: PBC_LP/κ-Carrageenan hydrogels (with KCl), H4: PBC_PP/κ-Carrageenan hydrogels (with KCl) : **(A)** percentage of porosity, **(B)** average pore size and **(C)** pore size in SEM image.

The interconnected pore structure of hydrogels is a critical factor in determining their functional performance, particularly in the domains of cellular penetration in tissue engineering and mass transport kinetics in drug delivery systems (Fan and Wang, 2017). The porosity of the prepared hydrogels (X1-H4) was measured, and the results are presented in Figure 4.

Figure 4A demonstrates the percentage of porosity of the hydrogel samples. The samples exhibited high porosity, ranging from approximately 66%–86%, indicative of the formation of an extensive, water-saturated network. This finding substantiates their suitability for biomedical applications. A comparative analysis revealed two main trends: the effects of PBC incorporation and ionic crosslinking. Initially, the hydrogels derived from PBC_LP and PBC_PP (H1 and H2) exhibited considerably higher porosity compared to the BC-based hydrogel (X1). This finding suggests the presence of the internal free space of the matrix, potentially by impeding the dense self-assembly of cellulose fibrils. Secondly, the incorporation of KCl as an ionic crosslinker resulted in a consistent reduction in porosity across all samples (i.e., X1 vs. X2). This phenomenon can be attributed to an increase in the polymer network’s crosslink density by the interaction of potassium ions (K^+^) with the sulfate ester groups (-OSO_3_^-^) of the κ-Carrageenan chain.

The average pore size of the hydrogel scaffolds was analyzed by measuring the pore size *via* SEM image (Figure 4C), and the results are represented as average values in Figure 4B. The results demonstrated a substantial enhancement in pore size refinement throughout the series, with a range of approximately 104 μm–216 μm. The non-crosslinked samples (X1, H1, H2) exhibited larger average pore sizes, indicative of a more extensive and loosely organized polymer network. The application of ionic crosslinking with KCl resulted in a shift of the microstructure towards consistently smaller pore diameters (i.e., X1 vs. X2)^45^. This refinement is attributed to the formation of stronger, localized bonding regions between the sulfate groups of kappa-carrageenan and K^+^ ions^46,47^, which reduces the distance between adjacent cellulose fibres. This increase in architectural density is directly related to the reduced porosity observed in Figure 4A.

### 3.4 Swelling behaviour

**FIGURE 5:**
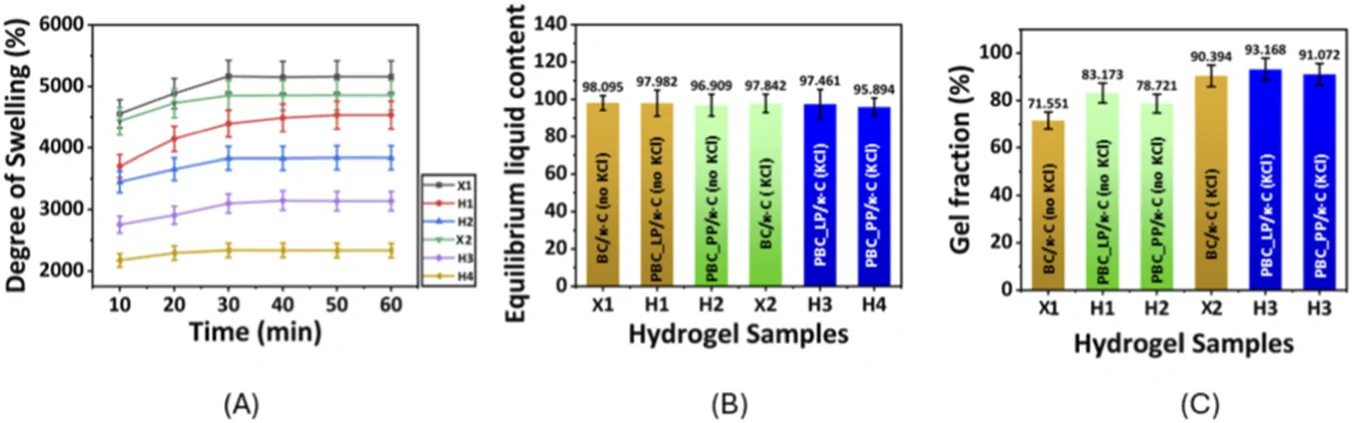
Swelling Study of BC/κ-Carrageenan, PBC_LP/κ-Carrageenan and PBC_PP/κ-Carrageenan hydrogels: **(A)** Degree of swelling, **(B)** Equilibrium liquid content (ELC) and **(C)** Gel fraction.

The swelling kinetics of the hydrogel samples presented in Figure 5A exhibit a classic diffusion-controlled process, characterized by a rapid initial adsorption and a subsequent plateau (Gehrke, 1993). It was observed that all samples attained a swelling equilibrium level within 30 min. The non-crosslinked samples (X1, H1, H2) showed greater swelling, which is directly related to their higher porosity and larger average pore diameter. This open-cell structure provides extensive free space for liquid retention. Conversely, the samples (X2, H3, H4) that employed K^+^ ions as crosslinkers demonstrated considerably constrained swelling. This reduction can be attributed to the elevated crosslinking density, in which the ionic interaction between K^+^ ions and the sulfate groups of the κ-Carrageenan matrix effectively curtails the mobility of the polymer chains (Qamar et al., 2024), thereby increasing the network’s structural rigidity.

The equilibrium liquid content (Figure 5B) was consistently high (over 95%) in all samples. The hydrogels’ high retention capacity indicates that they possess a highly hydrophilic three-dimensional matrix capable of retaining substantial amounts of aqueous medium. This property is essential for mimicking the ECM (Geckil et al., 2010).

### 3.5 Gel fraction and network stability

The gel fraction (Figure 5C) provides a quantitative assessment of network formation efficiency. The findings indicate that incorporating K^+^ ions as an ionic crosslinker consistently increases the gel fraction to a maximum of 93%. This increase indicates that the ionic crosslinking process effectively connects the polymer chains, reduces the soluble fraction of the components, and enhances the integrity of the hydrogel network (Nasution et al., 2022). The non-crosslinked samples (X1, H1, H2) exhibited low gel fraction, indicating a high tendency for polymer leaching in aqueous environments. Conversely, the elevated gel ratios observed in the crosslinked samples (X2, H3, H4) underscore the synergistic effect of the PBC matrix and ionic crosslinking in establishing a resilient, robust structure. This stability is paramount, as it ensures the hydrogel maintains its structural integrity and mechanical properties, even under conditions of substantial swelling and fluid retention.

### 3.6 *In vitro* cell viability assay

**FIGURE 6:**
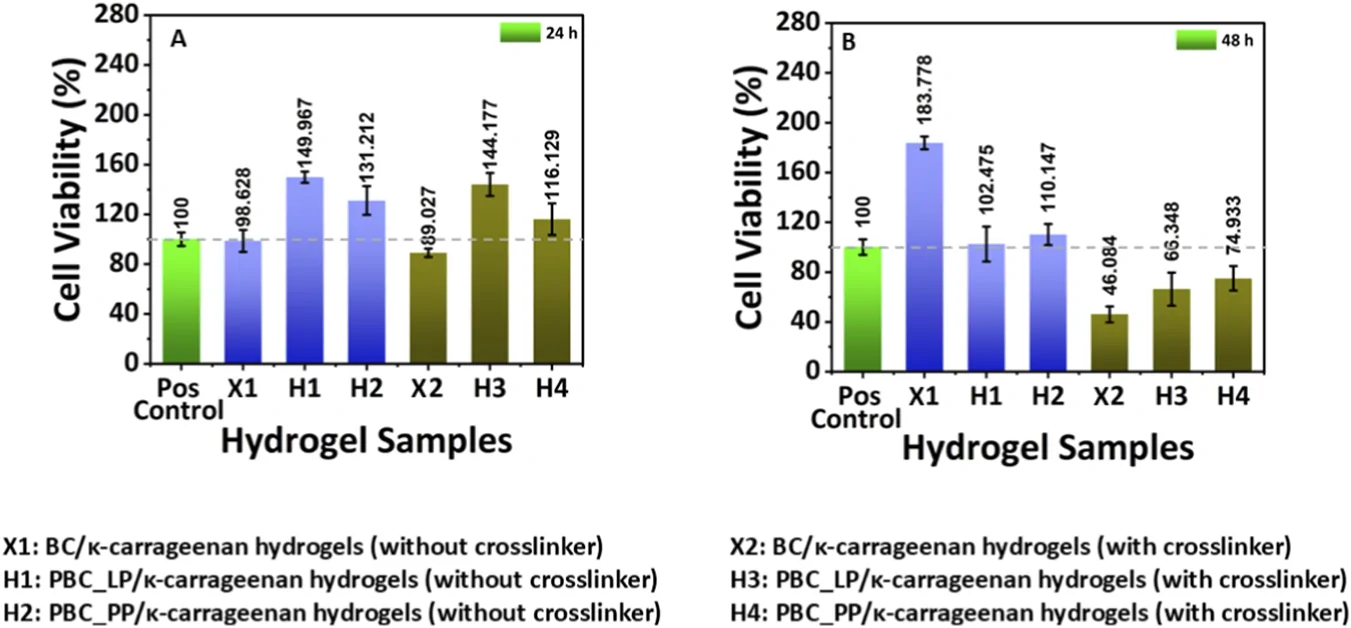
*In vitro* cell viability of BC/κ-Carrageenan, PBC_LP/κ-Carrageenan and PBC_PP/κ-Carrageenan hydrogels, where X1: BC/κ-Carrageenan hydrogels (without KCl), H1: PBC_LP/κ-Carrageenan hydrogels (without KCl), H2: PBC_PP/κ-Carrageenan hydrogels (without KCl) and X2: BC/κ-Carrageenan hydrogels (with KCl), H3: PBC_LP/κ-Carrageenan hydrogels (with KCl), H4: PBC_PP/κ-Carrageenan hydrogels (with KCl): **(A)** for 24 h and **(B)** for 48 h.

To assess the cell compatibility of the prepared hydrogels, cell viability was evaluated after 24 and 48 h (Figure 6). After 24 h, all non-crosslinked hydrogels (X1, H1, H2) exhibited viability equivalent to or greater than the positive control (100%), indicating favourable primary cell-component interactions. Specifically, H1 and H2 exhibited enhanced survival rates of approximately 149% and 135%, suggesting that the incorporation of PBC_LP and PBC_PP components fostered primary cell division or metabolic activity. Conversely, the crosslinked BC/κ-Carrageenan hydrogel (X2) exhibited a decline in cell viability rate, approximating 89%, suggesting a potential cytotoxic or inhibitory effect associated with the crosslinking process. Amongst the crosslinked systems, H3 exhibited relatively high viability (144%), while H4 demonstrated lesser viability (116%), suggesting that the nature and extent of PBC_LP and PBC_PP modification can mitigate the adverse effects of crosslinking.

At 48 h, more pronounced differences were observed between the crosslinked and non-crosslinked hydrogels. The non-crosslinked X1 hydrogel exhibited a substantial increase in cell survival, reaching approximately 138%, suggesting sustained cell proliferation and exceptional cytocompatibility. H1 and H2 exhibited survival rates that were comparable to or marginally higher than the control level, thereby substantiating the efficacy of promoting sustained cell proliferation. Conversely, all crosslinked samples (X2, H3, H4) exhibited lower viability than their non-crosslinked counterparts, with X2 demonstrating the lowest (46%). While H3 and H4 exhibited a modest enhancement over X2, their performance remained inferior to that of non-crosslinked systems. The decline in viability observed in crosslinked hydrogels may be attributed to several factors, including increased network density, impeded nutrient flow, or residual crosslinking agents (Kim et al., 2017). Conversely, non-crosslinked hydrogels offer a more conducive microenvironment, characterised by elevated porosity and enhanced cell permeability, thereby fostering metabolic activity and growth (Alheib et al., 2022).

The enhanced performance of H1, H2, H3, and H4 at 24 h suggests that PBC modification improves cytocompatibility. The results of this study demonstrate that while cross-linking improves structural stability, it also compromises biological performance. The non-cross-linked hydrogels with PBC_LP and PBC_PP demonstrate considerable cell compatibility and thus represent promising candidates for further development in tissue engineering applications. This result can be compared to the previous study (Bagheri et al., 2026), where the cell viability for the hydrogel samples ranges from >80% to 100%, making the hydrogels nontoxic for the fibroblast cells. The results in this study demonstrate that the hydrogels showed better cell viability at 24 h. Although the 48 h results show that hydrogels prepared without the KCl crosslinker have better biocompatibility, but the hydrogels with the KCl crosslinker are more cytotoxic.

### 3.7 Rheological properties

The rheological performance of BC/κ-carrageenan, PBC_LP/κ-carrageenan, and PBC_PP/κ-Carrageenan-based hydrogels was evaluated using strain- and frequency-sweep experiments to assess their viscoelastic networks and biomedical applications.

**FIGURE 7:**
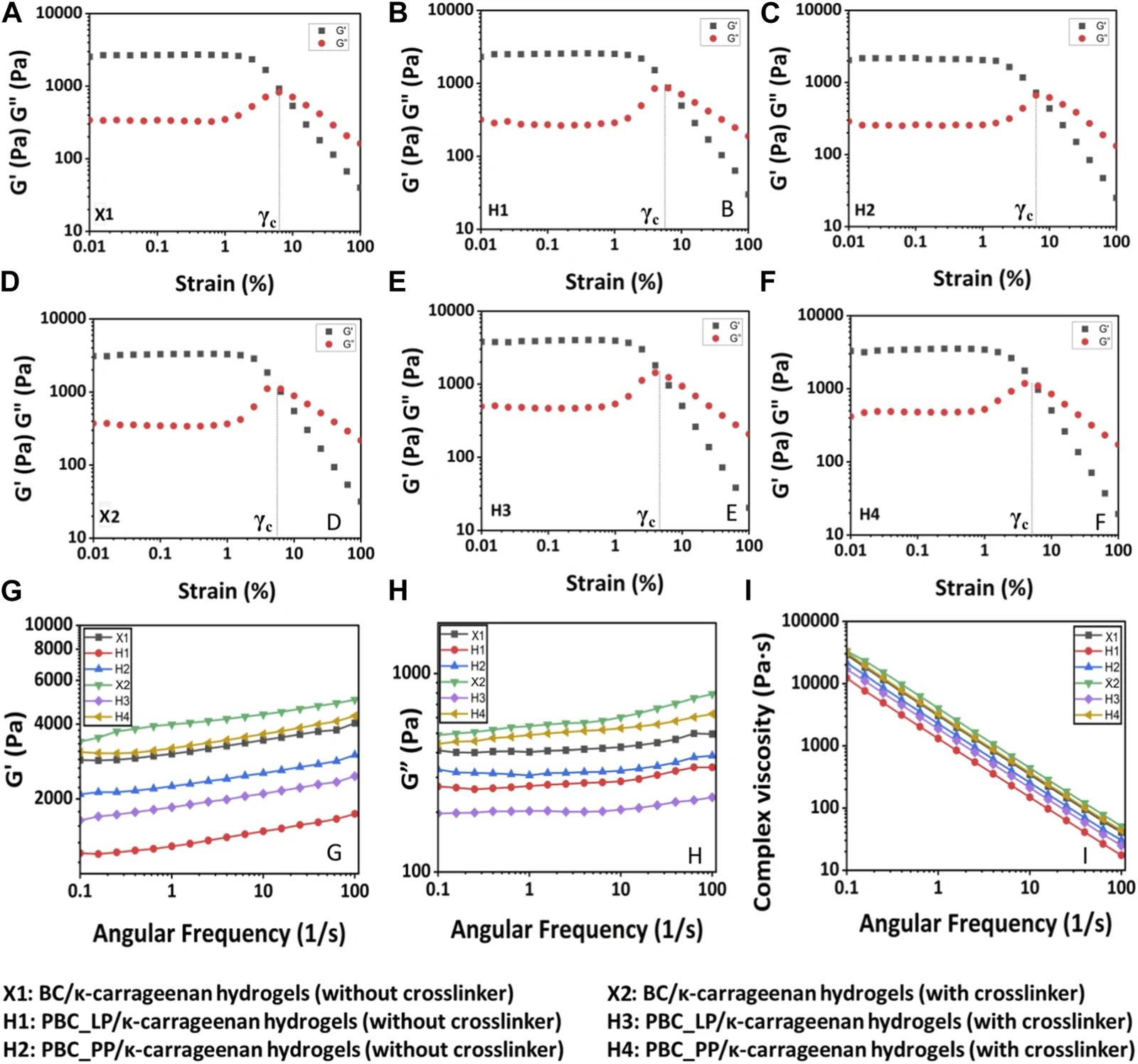
The rheological performance of BC/κ-carrageenan, PBC_LP/κ-carrageenan, and PBC_PP/κ-Carrageenan-based hydrogels; Strain sweep analysis **(A–F)** showing the LVE region and the dashed vertical lines indicate the gel-to-fluid crossover points (γ_c_), highlighting the strain-induced network disruption; frequency sweep analysis **(G–I)** comparing the storage modulus (G′), loss modulus (G″), and complex viscosity.

Strain sweep analysis (Figures 7A–F) demonstrated that all formulations exhibited a distinct linear viscoelastic region (LVE), wherein the storage modulus (G′) remained consistently greater than the loss modulus (G″). This finding indicates the presence of a predominantly elastic, gel-like structure. The observed crossover point (γ_c_), where G″ exceeds G′ at high deformation, indicates the end of the LVE region, indicating the onset of structural disruption of the hydrogel network. Following this point, the material progressively loses its elastic network because of network breakdown under increasing deformation (Middleman, 1995; Macosko, 1994).

Frequency sweep measurements (Figures 7G–I) further support these results, demonstrating that G′ and G″ remain frequency-dependent within the range that was tested. It is noteworthy that the incorporation of crosslinkers into samples X2, H3, and H4 resulted in substantially elevated G′ values compared to the non-crosslinked counterparts (X1, H1, H2). This observation confirms an increase in network density and stiffness. This mechanical property enhancement is further substantiated by the complex viscosity profile (Figure 7I), which manifests a discernible shear-thinning response in all specimens, wherein complex viscosity reduces with an amplified angular frequency. This tunable rheological profile suggests that the mechanical properties of these hydrogels can be precisely controlled by fabricating material compositions, particularly by adding PBC_LP and PBC_PP additives, to meet the biomechanical needs of specific tissue-engineering scaffolds (Reys et al., 2023).

### 3.8 Antimicrobial solution upload capacity and release profile

**FIGURE 8:**
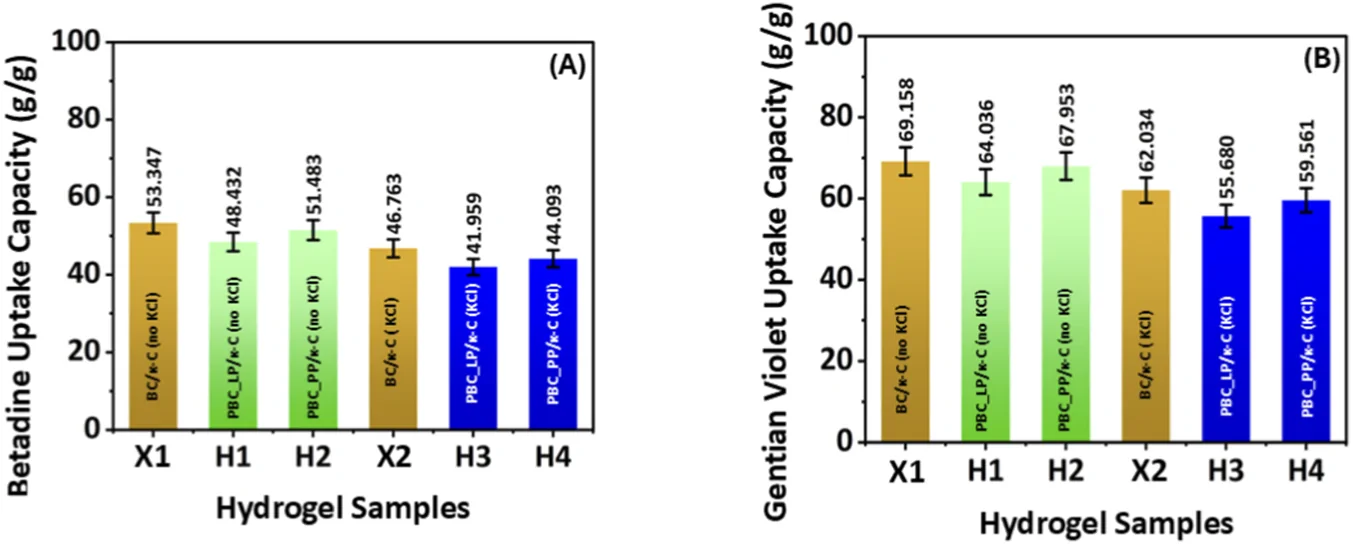
Antimicrobial solution (betadine **(A)** and gentian violet **(B)**) uptake capacity of BC/κ-Carrageenan, PBC_LP/κ-Carrageenan and PBC_PP/κ-Carrageenan hydrogel.

Figure 8 shows the antimicrobial solution uptake capacity of freeze-dried hydrogel samples for Betadine solution (Figure 8A) and GV (Figure 8B). The loading capacity for both Betadine and GV is higher in BC-based hydrogels than in PBC_LP- and PBC_PP-based hydrogels. Moreover, the uptake capacity of both antimicrobial solutions was reduced upon the addition of KCl as an ionic crosslinker. This reduction of loading capacity can be attributed to the more compact network structure of crosslinked hydrogels (X1, H1, H2) in comparison with non-crosslinked hydrogels (X2, H3, H4). Furthermore, the GV loading to the hydrogels is higher than the betadine loading for all the hydrogel samples.

**FIGURE 9:**
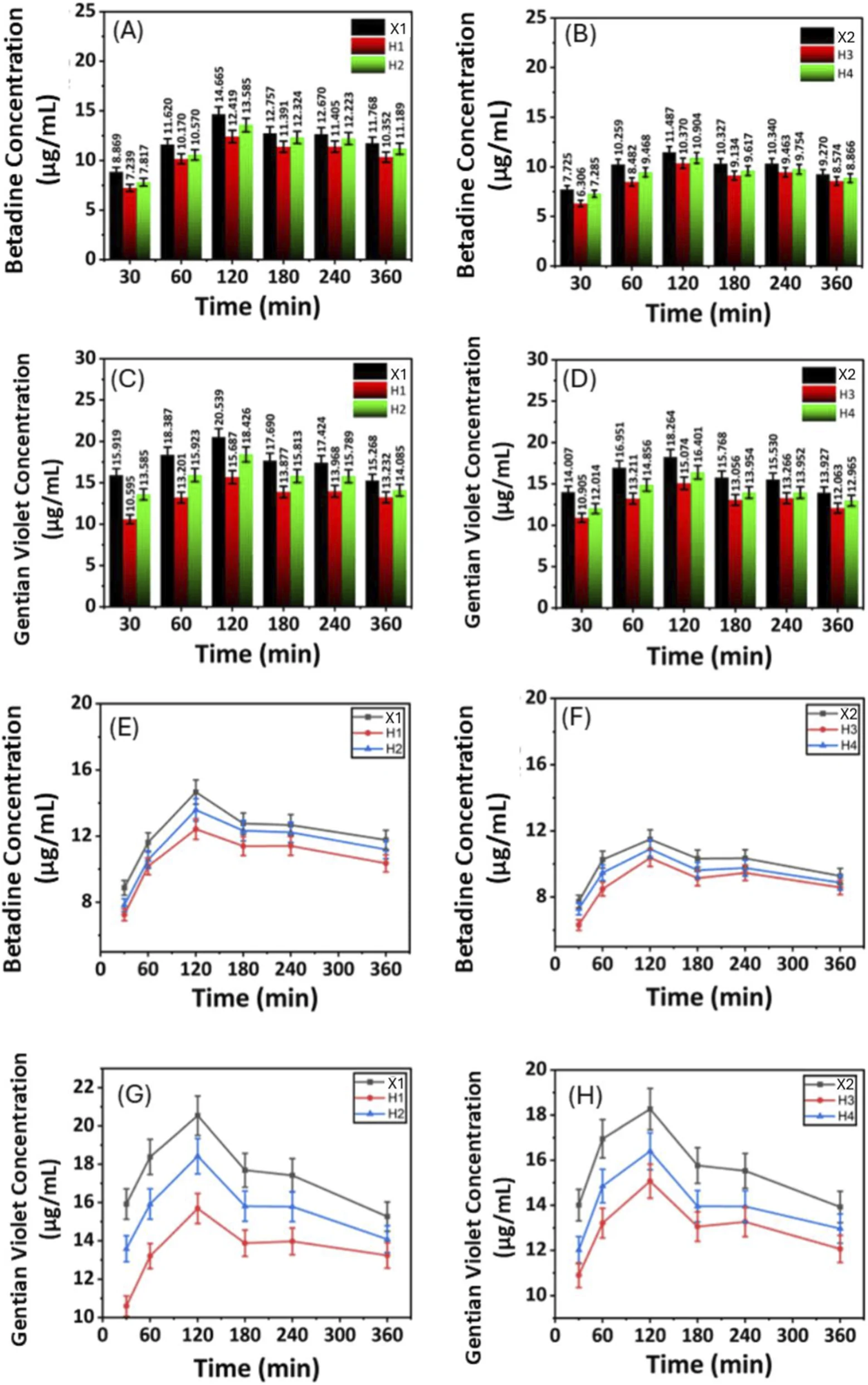
Antimicrobial solution (betadine **(A,B,E,F)** and gentian violet **(C,D,G,H)**) release profile of BC/κ-carrageenan (X1, X2); PBC_LP/κ-carrageenan (H1, H3) and PBC_PP/κ-carrageenan (H2, H4) hydrogels.

The release profiles of betadine and GV from BC/κ-carrageenan, PBC_LP/κ-carrageenan and PBC_PP/κ-carrageenan hydrogels are presented in Figure 9. All hydrogels exhibited a two-phase release pattern, consisting of an initial burst phase (30–120 min), followed by a slow, diffusion-controlled release up to 360 min. In the case of Betadine (Figures 9A,B,E,F), the non-crosslinked hydrogels demonstrated higher release than the crosslinked systems. The BC hydrogel (X1) demonstrated the most significant release (14–15 μg/mL at 120 min), while the PBC-containing hydrogels (H1, H2) exhibited moderately lower values. The release decreased significantly (10–11 μg/mL at 120 min) after crosslinking (X2, H3, H4), indicating that crosslinking limits drug diffusion by reducing the network pore size and increasing the matrix density (Rungrod et al., 2022). A comparable trend was observed for GV (Figures 9C,D,G,H), although its overall release was higher than that of betadine, reaching approximately 18–21 μg/mL in non-crosslinked hydrogels at 120 min. Crosslinked samples demonstrated a diminished yet more protracted release (16–17 μg/mL). The higher release of GV compared to betadine indicates weaker interactions with the polymer network and faster diffusion. In all systems, PBC-based hydrogels (H1-H4) exhibited consistently reduced release compared to the BC control, indicating enhanced drug retention. This phenomenon can be attributed to enhanced intermolecular interactions, such as hydrogen bonds, and to the denser microstructure introduced by the probiotic-derived material. Crosslinking played a significant role in regulating release profile, resulting in a slower, controlled release. The combination of an initial burst release and subsequent slow drug release is advantageous in wound healing, as it ensures rapid antimicrobial efficacy and sustained therapeutic effects.

### 3.9 Antimicrobial assay

**FIGURE 10:**
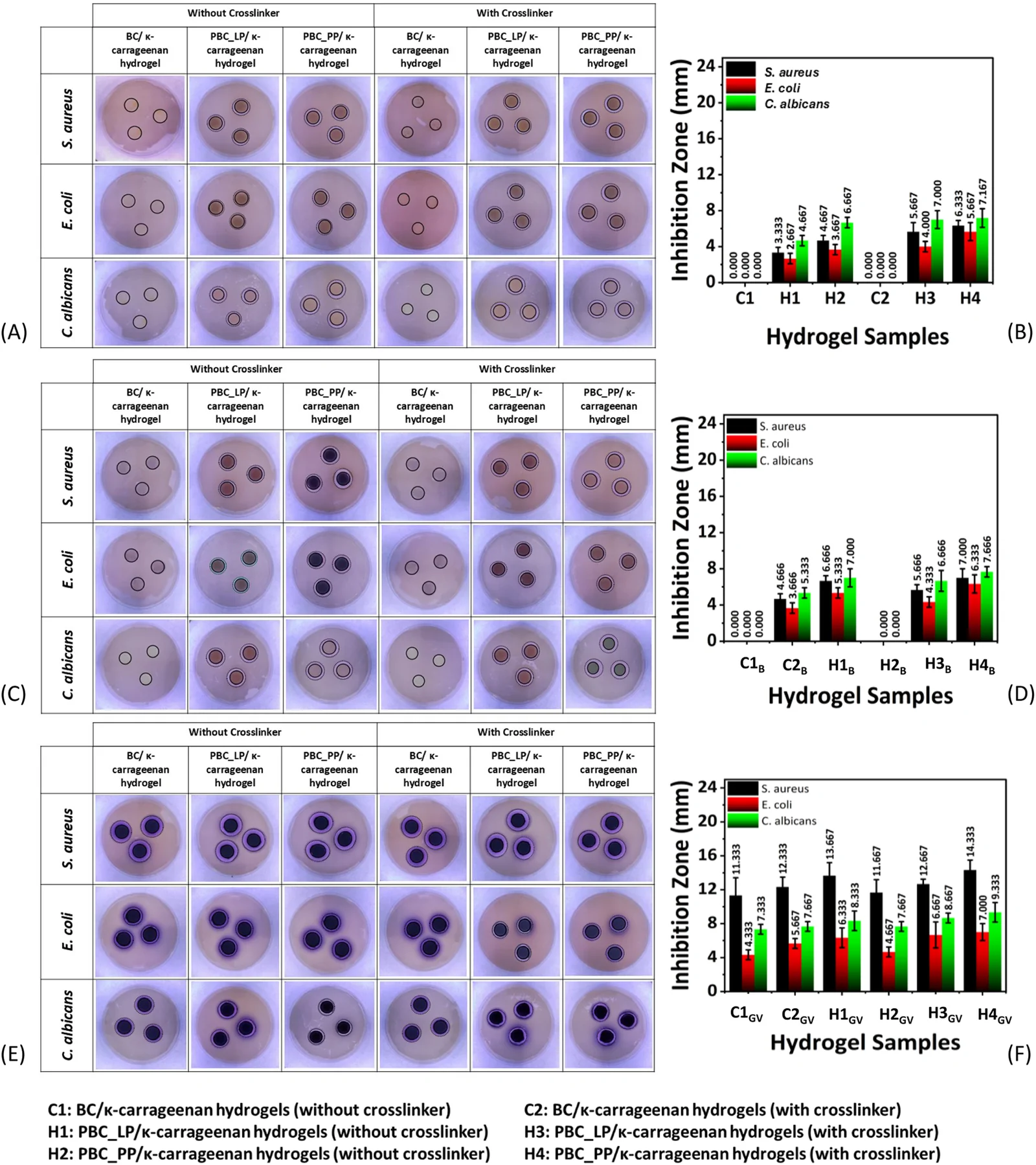
Antimicrobial assay of BC/κ-carrageenan, PBC_LP/κ-carrageenan and PBC_PP/κ-carrageenan hydrogels: visual image of zone of inhibition for **(A)** freeze-dried, **(B)** betadine soaked, **(C)** gentian violet soaked hydrogels and diameter of zone of inhibition for **(D)** freeze-dried **(E)** betadine soaked, **(F)** gentian violet soaked hydrogels.

Figure 10 demonstrates the antimicrobial effects of PBC_LP/κ-Carrageenan and PBC_PP/κ-Carrageenan hydrogels (freeze-dried, Betadine-soaked, and GV-soaked). The antimicrobial assay of PBC_LP/κ-Carrageenan and PBC_PP/κ-Carrageenan hydrogels (freeze-dried, Betadine-soaked, and GV-soaked) was performed to evaluate the antimicrobial activity of the hydrogels against pathogenic bacteria *E. coli* (Gram-negative) and *S. aureus* (Gram-positive), and fungi *C. albicans*, commonly present in the wound bed (Vindenes and Bjerknes, 1995; Binsuwaidan et al., 2023).

No inhibition zone was observed against any of the pathogenic strains for BC/κ-Carrageenan hydrogels, which were used as controls. However, both PBC_LP/κ-Carrageenan and PBC_PP/κ-Carrageenan hydrogels (experimental set) showed distinct inhibition zones (values shown in Table 3) for all the pathogenic strains. This result can be attributed to the presence of *L. plantarum* and *P. pentosaceus* in PBC_LP/κ-Carrageenan and PBC_PP/κ-Carrageenan hydrogels, respectively, as previous studies have shown that both probiotic bacteria exhibit inhibitory activity against all three aforementioned pathogens (Bajpai et al., 2016; Danilova et al., 2019). In the case of Betadine-soaked hydrogels, a similar result is observed, with increased inhibition zones for all the samples. The area of inhibition zones was highest against *C. albicans*, followed by *S. aureus*, and *E. coli* had the lowest. However, all the GV-soaked hydrogels had shown larger inhibition zones against all three pathogenic microbes. In this case, the largest inhibition zone is observed against *S. aureus*, followed by *C. albicans* and *E. coli*. This result suggests the effect of GV against all three pathogens (Pona et al., 2020). The diameter of the inhibition zones is demonstrated in Table 3.

**TABLE 3:** Zone of inhibition for BC/κ-Carrageenan (control set), PBC_LP/κ-Carrageenan (experimental set) and PBC_PP/κ-Carrageenan (experimental set) hydrogels against *Escherichia coli*, *Staphylococcus aureus* and *C. albicans*.

​	Pathogenic microbes	
Samples	*S. aureus*	*E. coli*	*C. albicans*
X1	0	0	0
H1	3.33 ± 0.57	2.66 ± 0.57	4.66 ± 0.57
H2	4.66 ± 0.57	3.66 ± 0.57	6.66 ± 0.57
X2	0	0	0
H3	5.66 ± 1	4 ± 0.57	7
H4	6.33 ± 0.57	5.66 ± 1	7.16 ± 1.04
X1_B_	0	0	0
H1_B_	4.66 ± 0.57	3.66 ± 0.57	5.33 ± 0.57
H2_B_	6.66 ± 0.57	5.66 ± 0.57	7 ± 1
X2_B_	0	0	0
H3_B_	5.66 ± 0.57	4.33 ± 0.57	6.66 ± 1.15
H4_B_	7 ± 1	6.33 ± 1	7.66 ± 0.57
X1_GV_	11.33 ± 2.08	4.33 ± 0.57	7.33 ± 0.57
H1_GV_	12.33 ± 1.15	5.66 ± 0.57	7.66 ± 0.57
H2_GV_	13.66 ± 1.52	6.33 ± 1.15	8.33 ± 1.15
X2_GV_	11.66 ± 1.52	4.66 ± 0.57	7.66 ± 0.57
H3_GV_	12.66 ± 0.57	6.66 ± 1.52	8.66 ± 0.57
H4_GV_	14.33 ± 1.15	7 ± 1	9.33 ± 1.15

## 4 Conclusion

In this study, different PBC/κ-Carrageenan composite hydrogels were successfully prepared using κ-Carrageenan and two different types of PBC (PBC_LP and PBC_PP), synthesized by *in situ* co-cultures of *K. xylinus* and *L. plantarum* and *P. pentosaceus,* separately. Both hydrogels were prepared with and without K^+^ mediated ionic crosslinking. This method enabled the investigation of the physicochemical, biological, and antimicrobial properties of the hydrogels of different compositions. FTIR and XRD analysis confirmed the successful integration of BC/PBC with κ-Carrageenan. Furthermore, the results demonstrated that the hydrogel formation occurred mainly through physical and ionic interactions, without altering the chemical identity of the constituent polymers. SEM analysis revealed highly porous, three-dimensional, interconnected structures, which are favourable for cell migration and nutrient transport.

The hydrogels exhibited high porosity, favourable swelling properties, and high equivalent liquid content, indicating good liquid absorption and moisture retention capabilities, which are essential properties for wound-healing applications. Ionic crosslinking of KCl increased the gel fraction and flow stability while concurrently reducing pore size and the degree of swelling. This resulted in hydrogel networks that were both mechanically stronger and structurally more stable. Furthermore, all hydrogels exhibited gel-like viscoelastic behaviour, suggesting their ability to be utilised for injectable or adaptive biomedical applications.

*In vitro* biocompatibility demonstrated that the addition of PBC significantly increased cell viability in comparison with BC-based control hydrogels. In particular, the non-crosslinked PBC_LP/κ-Carrageenan and PBC_PP/κ-Carrageenan hydrogels exhibited good cell viability, suggesting they might be potential candidate for tissue engineering and wound-healing applications. The hydrogels demonstrated efficient absorption and a biphasic release profile of the antimicrobial agents, namely, Betadine and GV. By contrast, the crosslinked systems exhibited more controlled release profile due to their dense polymeric networks. In particular, the PBC-containing hydrogels demonstrated inhibitory activity against *E. coli, S. aureus*, and *C. albicans*, a property absent in the BC-based control hydrogels. The inhibitory efficacy was further enhanced after loading Betadine and GV. The results of this study indicate that probiotic-derived components synergistically contribute to the *in vitro* antimicrobial activity of the composite system.

In conclusion, the developed PBC/κ-Carrageenan hydrogels exhibit a combination of beneficial structural, flowable, biocompatible, antimicrobial, and controlled-release properties, thus forming a single versatile platform. The study demonstrated that these hydrogels exhibited improved biological activity and antimicrobial efficacy compared to the control samples. Consequently, these hydrogels might be promising candidates for the development of advanced wound-healing and antimicrobial delivery systems. Despite the promising physicochemical characteristics, antibacterial activity, and *in vitro* cytocompatibility of the developed PBC/κ-carrageenan hydrogels, several limitations remain. The current findings are based on *in vitro* assessments, and further studies are required to evaluate *in vivo* wound healing efficacy, immune response, biodegradation behavior, and long-term stability. Future investigations involving animal models and clinical studies could facilitate further validation and standardization of these hydrogel systems.

## Data Availability

The original contributions presented in the study are included in the article/supplementary material, further inquiries can be directed to the corresponding author.
